# Topoisomerases inhibition and DNA binding mode of daunomycin–oligoarginine conjugate

**DOI:** 10.1080/14756366.2020.1780226

**Published:** 2020-06-19

**Authors:** Valeria Visone, Ildikó Szabó, Giuseppe Perugino, Ferenc Hudecz, Zoltán Bánóczi, Anna Valenti

**Affiliations:** a Institute of Biosciences and BioResources, National Research Council of Italy, Naples, Italy; b MTA-ELTE Research Group of Peptide Chemistry, Budapest, Hungary; c Department of Organic Chemistry, Eötvös Loránd University (ELTE), Budapest, Hungary

**Keywords:** DNA topoisomerase, topoisomerase inhibitors, daunomycin, DNA–protein interaction, circular dichroism

## Abstract

Cancer is a major health issue adsorbing the attention of a biomedical research. To fight this disease, new drugs are developed, specifically tailored to target biological pathways or peculiar components of the tumour cells. Particularly interesting is the use of intercalating agents as drugs capable to bind DNA and inhibit enzymes involved in DNA metabolism. Anthracyclines are the most commonly used anticancer drugs. In particular, daunomycin is used to cancer treatment by exploiting its ability to intercalate DNA and inhibit the activity of DNA topoisomerases implicated in the replication processes. Unfortunately, clinical application of anthracyclines is limited by their side effects. The conjugation with specific carriers could affect the selectivity and reduce side effect by improving stability and/or cellular uptake properties. We here report the biochemical characterisation of a daunomycin oligopeptide conjugate containing six residues of arginine, by the analysis of its fluorescence properties, DNA interaction and topoisomerases inhibitory effects.

## Introduction

Cancer is one of the leading global health problems worldwide with an increasing number of patients every year. The current oncotherapy methods include surgery, chemotherapy, radiotherapy, immunotherapy or their combination. The design and synthesis of new drugs for the treatment of cancer is a crucial area of research in medicinal chemistry^1–3^.

Anthracyclines are among the first chemotherapeutic agents developed and the most widely used, active against a large variety of tumours (leukaemia, lymphoma, cancer of the breast, lung, ovaries, etc.). In particular, daunomycin (Dau) and adriamycin exhibit the widest spectrum of antitumor activity against human cancers^4–7^. Despite the broad and relatively established usage of anthracyclines in antitumor therapy, their mechanism of action is still not fully understood.

These compounds are largely used in chemotherapy. However, their clinical application is limited by cardiotoxic side effects and the intrinsic or acquired drug resistance of tumour cells reducing the response to the treatment^8–10^. Due to the growing medical relevance of these compounds in anticancer chemotherapy, it is of great interest to improve their antitumor properties by structural modification. One of the most promising options is to design and prepare of bioconjugates with various oligo- and polypeptides as drug carrier^11–13^. Short oligomers containing five to eight residues of arginine (Arg_n_, where *n* = 5–8) are considered as cell-penetrating peptides capable to translocate covalently attached cargo into the cytosol^14–16^. Nevertheless, the nature of the carrier could influence the chemical and biological properties of conjugate. Therefore it is important to evaluate the specific contribution of the molecular transporter for each compound^12^^,^^16^^,^^17^.

**Figure 1.**
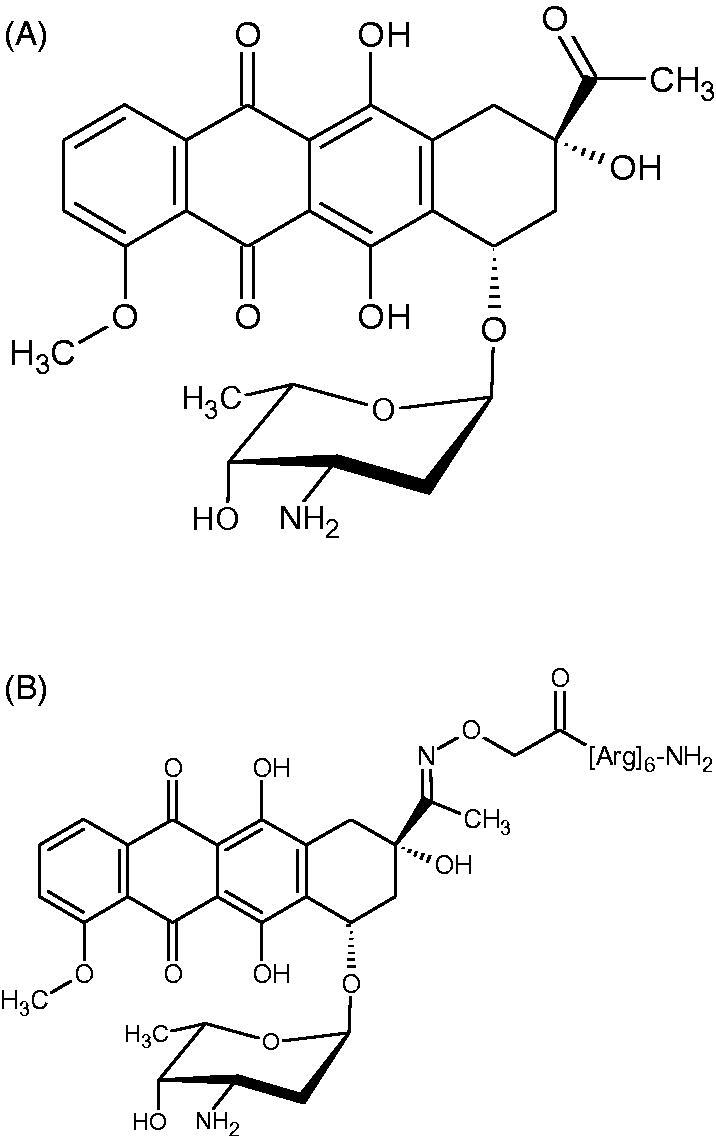
Chemical structure of daunomycin (A) and its Arg_6_ conjugate (B).

Previously, Miklán et al. described, for the first time, the synthesis of Dau-conjugate in which an oligoarginine peptide with 6 residues was attached to the drug by an oxime bond (Dau-Arg_6_, Figure 1(A,B)). It was demonstrated that Dau-Arg_6_ conjugate is stable under a broad range of pH and temperature conditions; retains the *in vitro* cytostatic effect on HL-60 and HepG2 cells and also it is taken up by murine and human tumour cell lines at higher level than that of the free drug^14^^,^^15^. Overall the previous data indicated that the peptide conjugate can efficiently deliver daunomycin into cells and that the conjugated showed a significant *in vitro* cytotoxic activity, suggesting that the oligoarginine conjugation represents a good avenue of the rational design strategy for improved antitumor agents, although no information about the effect of the arginine moiety on biochemical properties of the compound was known.

Dau affects a broad range of biochemical processes and different mechanisms have been proposed to be responsible for its cytotoxicity. These include the inhibition of DNA and RNA synthesis (mainly due to binding of the drug to the DNA) and Topoisomerase II (TopoII) poisoning (by trapping the enzyme at cleavage sites)^18^^,^^19^.

Topoisomerases normally solve the topological problems of DNA, which are generated during replication, transcription and recombination, by breaking (single or double strand) and rejoining the DNA strands^18^^,^^20^. Topoisomerase-active antitumor drugs interfere with the breakage and rejoining reaction of topoisomerases by trapping an abortive enzyme-DNA “cleavable complex”. TopoII has been identified as the primary target of anthracyclines. Specifically, Dau traps TopoII at breakage sites, stabilises the cleavage complex and impedes DNA resealing. Irreversible inhibition caused by Dau makes permanent double strands breaks, an essential factor for the consequent apoptosis in tumour cells^18^^,^^20^.

However, anthracycline-induced TopoII poisoning by trapping the enzyme at cleavage sites, is unlikely to be the only mechanism of cancer cell killing by anthracycline drugs.

A direct relationship between affinity towards DNA and biological activity has been established for various anthracycline derivatives suggesting that the DNA binding play a crucial role in the drug effectiveness. Several lines of evidence have demonstrated that Dau inhibits the activity of Type I topoisomerases, essential enzymes in higher eukaryotes, required to relax DNA supercoiling generated by transcription, replication and chromatin remodelling^20^^,^^21^. These classes of enzymes cut one strand of double stranded DNA, relax the strand, and re-strengthen the strands. In eukaryotes, topoisomerase I poisons (i.e. camptothecin and its derivative) exert their anticancer effects by trapping TOP1-DNA covalent complexes. Differently, Dau suppresses TopoI activity as well as other DNA processing protein and enzymes by interfering with their DNA binding, suggesting that anti-cancer activity of Dau is also attributable to a general protein inhibition^20–23^.

Previous data demonstrate that the attachment of an oligopeptide affect DNA binding property of Dau, suggesting that the presence of the peptide could be inhibitory for this compound^12^. Staring from these observations we were interested in studying the contribution of the oligoarginine part on the biochemical properties of this Dau-conjugate, in comparison with the free drug, evaluating the DNA–drug interaction, fluorescence properties, and enzymes inhibition.

Our data demonstrate that Dau-Arg_6_ retains the biochemical properties of Dau: inhibits the activity of human TopoII (h-TopoIIα), binds the DNA mainly by intercalation and stabilise the double helix similarly to the free drug. In addition, we found that oligoarginine affect drug-DNA interaction suggesting that this oligopeptide may play a general role in in protein/enzyme inhibition.

## 2. Materials and methods

### 2.1. Materials

For peptide synthesis amino acid derivative and Rink Amide 4-methylbenzhydrylamine (Rink Amide MBHA) resin were purchased from IRIS Biotech GmbH (Marktredwitz, Germany). Reagents for synthesis: *N*,*N*’-diisopropylcabodiimide (DIC), 1-hydroxybenzotriazole (HOBt), 1,8-diazabicyclo-[5.4.0]undec-7-ene (DBU), piperidine, phenole, thioanisole, triisopropylsilane (TIS), daunomycin (Dau), Boc-(aminooxy)acetic acid (Boc-Aoa-OH), and hydroxylamine hydrochloride were purchased from Sigma Aldrich (Budapest, Hungary). Trifluoroacetic acid (TFA) and, solvents for the synthesis as well as HPLC grade acetonitrile were obtained from Molar Chemicals (Budapest, Hungary).

Calf thymus DNA (catalogue number D1501) was purchased from Sigma and dissolved in 10 mM Tris-HCl, pH 7.5 at final concentrations of 1 mg/ml. The reconstituted solution was stored at −20 °C until experiments. Daunomycin and its Arg_6_-derivative were prepared as previously reported^14^ with some modifications as described below and kept at 4 °C.

Human topoisomerase IIα (h-TopoIIα) was purchased from Affymetrix (product number 78303) and stored at −20 °C. Human Topoisomerase I (Sigma, St. Louis, MO). pBluescript (pBs) negatively supercoiled plasmid was directly extracted from an over-night *E. coli* cell culture by Invitrogen HiPure Plasmid Midi prep Kit according to the instruction of the manufacturers.

### 2.2. Synthesis of Dau-Arg_6_ conjugate

Synthesis of Dau-Arg_6_ conjugate was performed as described by Miklan et al.^14^ with some modifications. Briefly, the synthesis of hexaarginine oligopeptide was carried out manually on Fmoc- Rink-Amide MBHA resin using standard Fmoc/*^t^*Bu strategy. For coupling, Fmoc-Arg(Pbf)-OH and DIC/HOBt as coupling agents, were applied in DMF for *in situ* active ester formation. The N-terminal of the hexaarginyl-resin was functionalised by Boc-(aminoxy)acetic acid (Boc-Aoa-OH), using DIC/HOBt coupling reagents. The aminoxyacetylated peptide (Aoa-Arg_6_) was cleaved from the resin with TFA in the presence of scavengers (phenole, thioanisole). It should be noted that EDT was replaced by TIS and hydroxyl amine.

Before the daunomycin conjugation, the crude product was purified by semipreparative Knauer RP-HPLC system (H. Knauer, Bad Homburg, Germany) on a Phenomenex Luna C_18_ column (5 µm, 100 Å, 250 × 4.6 mm I.D.) (Torrance, CA), as stationary phase. Linear gradient elution was applied using eluent A (0.1% TFA in water) and eluent B (0.1% TFA in ACN-water (80:20 v/v%)). The purified Aoa-Arg_6_ peptide was characterised by analytical RP-HPLC and ESI-MS (Figure 1). The Aoa-Arg_6_ peptide was dissolved in 0.2 M NaOAc buffer (pH 5.2) at a peptide concentration of 10 mg/ml, and 50% excess of Dau·HCl was added to the solution. Oxime ligation was carried out overnight. In order to separate efficiently the conjugates from the unreacted free drug instead of the previously used FPLC, RP-HPLC was applied and the excess of Dau was reacted with hydroxylamine hydrochloride to increase the difference in retention time of the compounds. The purified conjugate (Dau-Aoa-Arg_6_) was dissolved in distilled water, freeze-dried, and was characterised by analytical RP-HPLC (R_t_: 32.3 min) and ESI-MS (M_av_ calc/M_av_ meas: 1536,7/1537.2).

### 2.3. Topoisomerases assay

DNA relaxation assays were performed as previously described^24^^,^^25^ using the negative supercoiled pBluScript plasmid (pBS) purified from *Escherichia coli* as a substrate of DNA topoisomerases.

For h-TopoII-α assay the enzyme (0.4 units) was incubated for 30 min at 30 °C with negatively supercoiled pBS plasmid (7.5 nM), in a final volume of 20 µl of 1× reaction Buffer (10 mM Tris-HCl, pH 7.9, 50 mM NaCl, 50 mM KCl, 0.1 mM EDTA, 5 mM MgCl_2_, 15 µg/ml BSA, 1 mM ATP).

For h-TopoI assay the enzyme (100 nM), was incubated for 60 min at 37 °C with negatively supercoiled pBluescript plasmid (7.5 nM) in a final volume of 20 µL of 1X reaction Buffer (10 mM Tris-HCl, pH 7.0, 100 mM NaCl, 0.1 mM NaEDTA, 10 mM MgCL_2_ and 50 µg/ml BSA).

To test the effect of Dau or Dau-Arg_6_ on Topoisomerases activity, the reactions were incubated for 10 min at 4 °C to ensure binding equilibrium, afterward the topoisomerases were added and incubated as described above.

All reactions were stopped by the addition of SDS to a final concentration of 1% and the products were analysed by electrophoresis on a 1.2% agarose gel in presence of 0.5X Tris-Borate EDTA buffer. To visualise the reaction products, the gels were stained post-electrophoresis by soaking in a bath containing ethidium bromide (1 µg/ml) for 10 min at room temperature and destained in deionised water. Finally the DNA bands were visualised using a BioRad Versadoc imaging system and quantified by Quantity One® software. The relative intensity of each band in the experiment was measured and used to calculate the amount of total DNA and the fraction of total products. For each condition the relaxation activity was expressed as a percentage of DNA products on total DNA^26^.

### 2.4. Fluorescence spectroscopic studies

Fluorescence measurements of Dau and Dau-Arg_6_ were carried out with a JASCO spectrofluorimeter (FP8200) using Quartz cells of 1 cm. Spectra were recorded at drug concentration of 10 µM in a final volume of 1 ml of 20 mM Tris buffer (pH 7.5).

For quenching experiment fluorescence titrations were conducted by keeping the concentration of drugs constant and varying the concentration of pBs plasmid from 5 to 40 nM. The spectroscopic measurements were recorded after 5 min to reach the equilibrium between the drugs and DNA. The spectra were recorded at room temperature in the wavelengths range of *λ* = 500–700 nm.

### 2.5. Circular dichroism analysis

Circular dichroism (CD) measurements were performed on a JascoJ-810 spectropolarimeter using a 0.1 cm path-length quartz cuvette at room temperature and the following parameters: three scans at a speed of 20 nm/min, 3 accumulations, 4 s time constant, 1 nm band width and 0.2 nm data pitch^25^.

The spectral bandwidth was set up at 1 nm in the wavelength range of *λ* = 225–300 nm. The CD spectra of Calf Thymus DNA (ctDNA; 0.2 mg/mL) with or without drug (at selected concentrations) were measured under a nitrogen atmosphere using a 10 mM Tris-HCl buffer at pH 7.5. Results are presented as a mean of three scans and the buffer background was electronically subtracted from each measurement.

ICD measurements were recorded by keeping the concentration of ctDNA constant (0.2 mg/mL) and increasing drugs concentrations from 0 to 120 µM.

### 2.6. Melting studies

Melting studies were performed on a JascoJ-810 spectropolarimeter as described above by raising the temperature from 5 to 90 °C in 1 °C/min increments using a circulating water bath and monitoring the profiles at *λ* = 260 nm.

The DNA thermal melting was measured by monitoring the changes of HT[V] signal with temperature. Melting temperatures (*T*_m_) were determined for each transition data by fitting sigmoidal curves using Boltzmann equations and obtaining good *R*-squared values (>0.95).

## 3. Results and discussion

### 3.1. DNA topoisomerases inhibition

**Figure 2.**
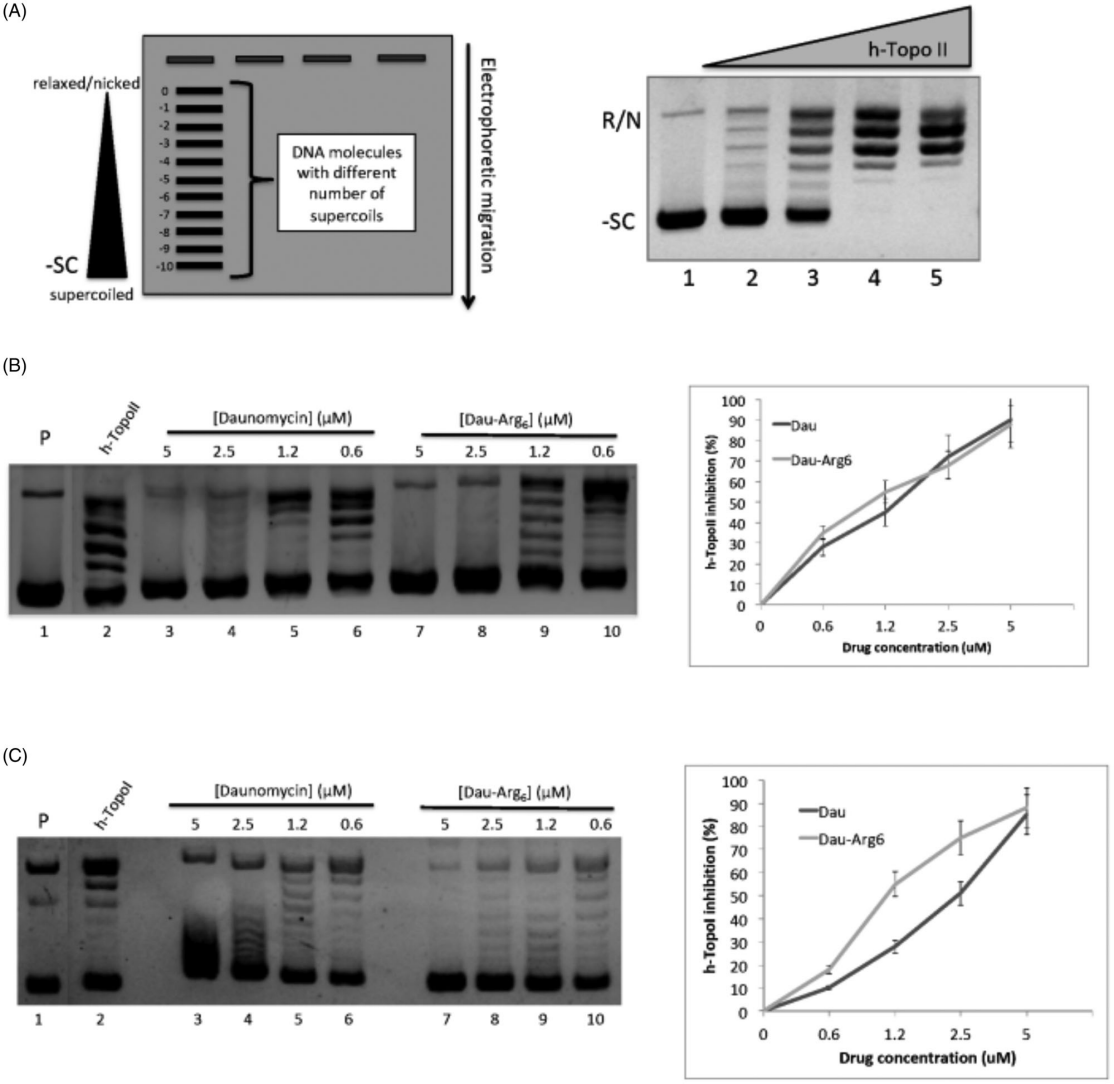
Topoisomerase assay. (A) Left panel: schematic representation of gel migration of the topoisomers (plasmid molecules with different supercoiling degree) from negative supercoiled (-10) to relaxed form (0). Right panel: the gel shows plasmid relaxation catalysed by h-TopoIIα: DNA Plasmid was incubated without (lane 1) or with (lanes 2–5) increasing concentrations of h-TopoIIα. (B) Inhibition of plasmid relaxation by Dau and Dau-Arg_6_: the left panel shows h-TopoII activity in absence (lane 1) and in presence of decreasing concentrations of Dau (lanes 3–5) or Dau-Arg_6_ (lanes 7–10); lane 1: plasmid alone. Right panel shows the Quantification of h-TopoII inhibition as a function of drugs concentration. The relative intensity of each band in the gel was measured and used to calculate the amount of total DNA (the unprocessed DNA plus the distinct topoisomer species), and that of all individual reaction products. For each condition, the inhibition of topoisomerase activity is expressed as a percentage of the produced topoisomers over the total DNA in each lane. Error bars represent standard deviations of three independent assays. (C) Inhibition of plasmid relaxation by Dau and Dau-Arg_6_: the left panel shows h-TopoI activity in absence (lane 1) and in presence of decreasing concentrations of Dau (lanes 3–5) or Dau-Arg_6_ (lanes 7–10); lane 1: plasmid alone. Right panel shows the quantification of h-TopoI inhibition as a function of drugs concentration. The inhibition was determined as described in (B).

Dau is a catalytic inhibitor of TopoII. By forming a drug–enzyme–DNA complex, the drug prevents the relegation step normally catalysed by topoisomerase inhibiting the relaxation activity^19^. The principal approach to evaluate the topoisomerases activity is based on the different electrophoretic mobility of the relaxed DNA molecules (products) compared with the negatively supercoiled (substrate). The gel electrophoresis in Figure 2(A) (right panel) shows the DNA relaxation of negative supercoiled plasmid by h-TopoIIα resulted in a range of products that migrate slower than the supercoiled one. To understand the contribution of hexarginine moiety of the conjugate on h-TopoIIα inhibition, we evaluated the effect of Dau-Arg_6_ in comparison with free drug (Figure 2(B), left panel). Quantitative analysis shows that, as expected, daunomycin inhibited DNA relaxation (at concentration of 1.2 μM inhibits 50% of the total h-TopoIIα activity; Figure 2(B), right panel). Interestingly, similar results were observed for Dau-Arg_6,_ suggesting that the presence of the peptide does not influence the mechanism of h-TopoIIα inhibition.

Several lines of evidences reported that the Dau also inhibits type I topoisomerases (TopoI); however, the mechanism underlying the inhibition is not mediated to covalent complex stabilisation, but it occurs by preventing DNA binding^22^^,^^23^. We tested TopoI activity in presence of either Dau or Dau-Arg_6_: surprisingly, the inhibitory effect of Dau-Arg_6_ is greater than the free drug, resulting as 50% inhibition of the TopoI activity at not saturating concentration (i.e. 1.2 uM), whereas the Dau blocks the activity only for 20% (Figure 2(C)). This result suggest that the peptide also contributed to TopoI inhibition probably interfering with enzyme–DNA interaction

### 3.2. Fluorescence emission quenching studies

In addition to Topoisomerases inhibition, the antitumor activity of daunomycin is also associated with its ability to intercalate DNA^26–29^. We then wondered whether oligoarginine affect the DNA binding mode of Dau.

**Figure 3.**
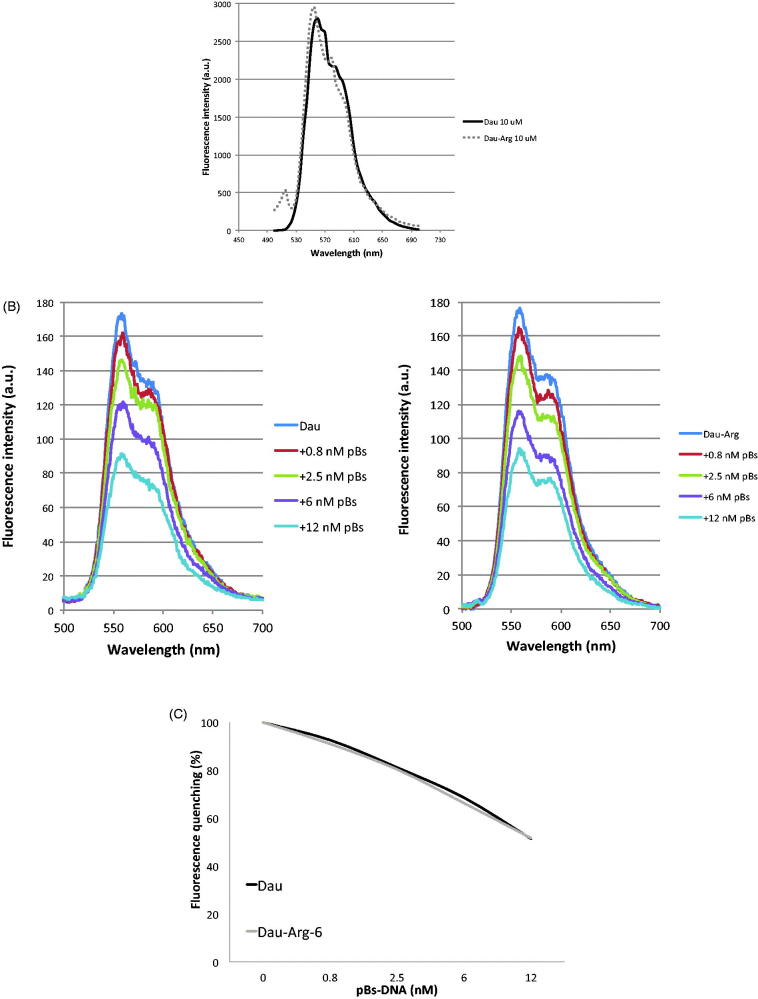
(A) Emission spectra of Dau and Dau-Arg_6_ derivative. (B) Effect of DNA plasmid on Fluorescence emission. (C) The chart shows the percentage of fluorescence quenching in function of plasmid concentrations.

The fluorescence characteristics of anthracycline are often used to assess interaction of the drug with DNA and other macromolecules. It is known that when the drug interacts with DNA, fluorescence may change depending on the impact of such interaction on the drug conformation, or *via* direct quenching effect^29^^,^^30^. We examined the fluorescence spectra of Dau and Dau-Arg_6_ at *λ* = 500–750 nm showing, as expected, the same trend with an emission maximum at *λ*_max_=596 nm for both drugs (Figure 3(A)). Thus, we evaluate the effect of DNA plasmid (pBs) on fluorescence spectra. Titration equilibrium studies were performed by adding increasing amounts of DNA plasmid to a fixed amount of each drug, then measuring the fluorescence emission after equilibrium. The emission spectra reveal that fluorescence intensity of both drugs decreases with increasing concentration of DNA, indicating a fluorescence quenching after DNA binding (Figure 3(B)). The quantitative analysis shows the percentage of fluorescence quenching in function of DNA concentrations indicating the same trends for Dau and its derivative (Figure 3(C)).

### 3.3. Melting studies

**Figure 4.**
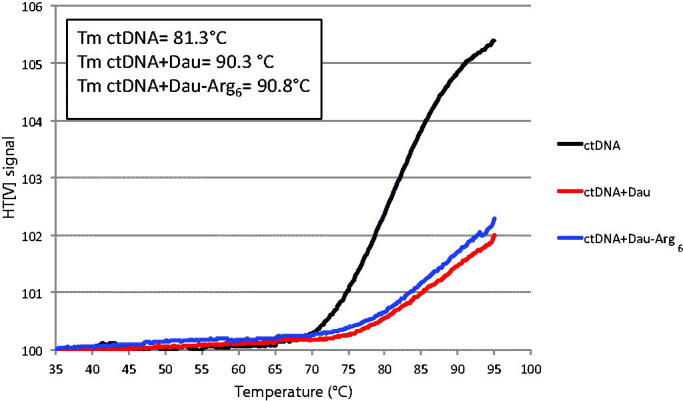
Melting analysis. Thermal denaturation of ctDNA (0.2 mg/mL) at *λ* = 260 nm in the absence and the presence of 4 µM Dau and Dau-Arg_6_ in Tris–HCl buffer. The melting temperatures (*T*_m_) were determined as described in Section 2 and reported in the inset.

Thermal behaviours of DNA in the presence of ligands/drugs give information about the DNA conformational changes when the temperature is raised, and they can also predict about the strength of interactive forces existing between drug and DNA^31^^,^^32^. The melting temperature (*T*_m_) is defined as that temperature that 50% of the DNA is double stranded and 50% is single stranded. This value reflects the intrinsic stability of nucleic acids (% of CG) and the extrinsic contributions from the solution, such as ionic strength, small molecules, and DNA intercalators. If a drug interacts with DNA stabilising its duplex form, a characteristic increase in the *T*_m_ is observed during thermal denaturation. Circular dichroism (CD) spectroscopy can be used to monitor thermal denaturation of DNA: we determined the *T*_m_ of Calf Thymus-DNA (ctDNA) in the presence of these compounds (Figure 4). Under the conditions used, the melting profile of ctDNA without drugs showed a temperature dependent base pairs opening with a *T*_m_ of 81 °C. The binding of the drugs to DNA resulted in a significance increase in melting temperature by about 9 °C, suggesting an intercalative binding mode. Taken together, these comparative studies demonstrate that both Dau-Arg_6_ conjugate and the free drug are able to interact and stabilise dsDNA.

### 3.4. Circular dichroism analysis and DNA conformation

**Figure 5.**
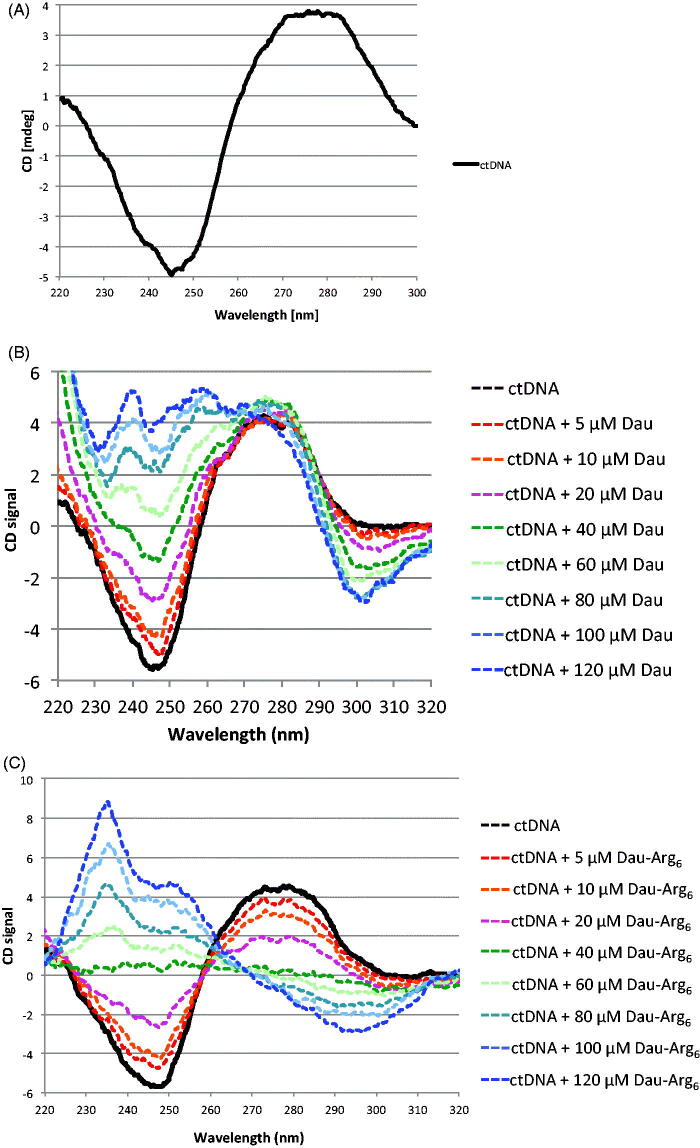
A. Circular dichroism analysis of Ct-DNA. (B) Circular dichroism analysis of ctDNA shows a typical features of B-DNA: a positive band at *λ* = 275 nm due to the base stacking and a negative band at *λ* = 245 nm specific of DNA helicity. (C) CD spectra of ctDNA in presence of increasing concentrations of Dau and Dau-Arg_6_ showing the changes in CD signals of B-DNA, after drugs binding.

Circular dichroism is widely employed in the studies of nucleic acids structures and to monitor conformational polymorphism of DNA. Due to its accuracy and sensitivity, this technique can be also used for an in deep analysis of drug–DNA interaction: the changes in CD signals of B-DNA, after drugs binding can be assigned to the corresponding changes in DNA morphology^26^^,^^33^^,^^34^. To analyse the interaction mode between drugs and DNA, we performed CD analysis using ctDNA. As expected, the CD spectrum of ctDNA shows a typical feature of B-DNA: a positive band at *λ* = 275 nm due to the base stacking and a negative band at *λ* = 245 nm specific of DNA helicity (Figure 5(A)). It is known that interaction between DNA and molecules induces variation in these bands. Specifically, whereas binding to DNA grooves results in little or no changes of the base stacking and helicity bands, intercalation of molecules into the double helix induces a noteworthy change of intensity of the bands^35^. It is reported that upon addition of the anthracyclines to DNA, the spectrum changes occurring in the range of *λ* = 320–200 nm, are related to structural modification of the nucleic acid^36^. In our hands, upon the addition of Dau or Dau-Arg_6_, the CD spectra of ctDNA showed a changes of negative band at *λ* = 245 nm (shifting to zero level) in a concentration dependent manner, indicating that the drugs modify DNA structure by helix intercalation (Figure 5(B,C)). Surprisingly, only the Dau-Arg_6_ induces a strong decrease also of the positive band at *λ* = 275 nm in a concentrations dependent manner, indicating a perturbation of the bases stacking interactions (Figure 5(C)). It is reported that the DNA spectrum in presence of Arg_6_ exhibits a decreased intensity of peak at *λ* = 275 nm as a function of arginine concentration^36^^,^^37^ supporting that electrostatic binding of ions is involved in the interaction between arginine residues and DNA.

Finally, the CD spectra analysis also showed the appearance of a negative peak at *λ* = 300 nm characteristic of an Induced Circular dichroism (ICD). Many DNA binding ligands are achiral and so optical inactive. However, through interaction with DNA, these ligands can acquire an induced CD signal. In particular a positive ICD peak is due to groove binding and a negative ICD peak is due to intercalative binding^38^^,^^39^. The ICD spectra of both drugs are almost identical, and show a negative peak at *λ* = 300 nm confirming the DNA intercalation.

CD studies demonstrate that Dau as well as its oligopeptide conjugate interact with DNA mainly by intercalation. Furthermore, because of positive charges of the Arg_6_ on the molecule, the conjugate drug possesses a natural tendency to be attracted by the negative DNA and extending contacts with this macromolecule.

## 4. Conclusion

Daunomycin is widely used in cancer chemotherapy. Despite its usage is well established, serious side effects limited its involvement in antitumor therapy^7^^,^^8^. Further development of its efficacy could cover the synthesis of analogues and conjugates for tumour cells targeting. It has been reported that oligoarginine containing five to eight arginine residues are considered as cell-penetrating peptides capable to translocate covalently attached cargo into the cytosol. The analysis of cell-penetrating properties is in the focus of intense research, but the mechanism by which these compounds enter the cells is still not completely clear^40^^,^^41^.

The *in vitro* cytotoxicity as well as the internalisation ability of the Dau-Arg_6_ conjugate studied earlier^14^ clearly demonstrated that the peptide conjugate can efficiently deliver daunomycin into cells and the conjugate exhibited substantial *in vitro* cytotoxic activity. Considering that free Dau intercalates DNA and inhibits topoisomerase activity^22^^,^^42^ we were interested in studying the effect of the attached (conjugated) (Dau-Arg_6_) unit on these functions. Previously we investigated the effect of other oligopeptide on the DNA binding property of Dau, and it turned out that the presence of a peptide could be inhibitory^12^. Differently our finding presented in this paper indicate that the presence of the cell-penetrating peptide (Arg_6_) does not affect DNA binding as well as its ability to inhibits topoisomerases suggesting that the compound could be used as delivery agent.

Using biochemical and spectroscopic techniques, we examined, the biochemical properties of an oligoarginine peptide conjugate in which six arginine residues (Arg_6_) was conjugated with Dau on double helical structure of DNA. Topoisomerases assays show that Dau-Arg_6_ conjugate inhibits the h-TopoIIα in a concentration-dependent manner similarly to the free drug (Dau). Surprisingly, the inhibition of TopoI increases when oligoarginine is attached to the drug, suggesting that the peptide makes the Dau a better inhibitor of this enzyme, likely by a direct interaction with DNA.

Thus, we focussed our attention on the analysis of DNA-drug interaction. Different methods were used to investigate the interaction mechanism between drug and DNA. Fluorescent quenching assay confirms that the Dau-Arg_6_ interact with DNA. In addition, the CD spectra analysis showed a structural distortion of the canonical B-conformation upon binding with the drugs, indicative of an intercalative binding mode (as already observed for Dau^29^^,^^43^. Interestingly, Arg_6_-moiety also induces conformational modifications implying that an electrostatic interaction also occurred. These finding suggest that the intercalative binding mode of Dau together with a “*charges effect*” of oligoarginine residues, may play a synergic role on protein/enzyme inhibition improving the drug effectiveness. These observations are in line with the experimental data of TopoI inhibition revealing an increased inhibitory effect of Dau-Arg_6_ compared with the free drug. Electrostatic interactions between the negative phosphate and the positive charge present in the Arg_6_-moiety are relevant for DNA groove binding and the presence of protonated groups improves the attraction for DNA grooves under physiological conditions^44^. Taken together we propose the following hypothesis: Dau-Arg_6_ conjugate works as a better TopoI suppressor since this compound intercalates into DNA by the Dau portion, induces a DNA structure distortion by the Arg_6_ part, then inhibits the binding of DNA to TopoI, and finally affects the catalytic activity of topoisomerase. The modification of chemical structure in several anticancer drug candidates is crucial for determining their affinity for DNA and for the inhibitory activity to crucial enzymes in cells’ vital processes by optimising anticancer activity separately or synergistically^44^.

Discovery of new anticancer chemotherapeutics capable of interacting with DNA and of inhibiting Topoisomerase enzymes is highlighted in anticancer research. The results indicate that DNA binding and enzymes inhibition are widely explored mechanisms and most promising in the proposition of new anticancer candidates as well as in drugs repurposing^45^. In this way, synthesis of new compounds is optimised by the junction with portions that have DNA affinity by intercalation and/or groove binding to increase the efficiency at low concentrations of said drugs. The results of biochemical studies presented here could represent a proof-of-concept, opening to new strategies in order to expand the effect of anthracycline on the inhibition of DNA–enzyme interaction, and could contribute to the rational design of new chemotherapeutic compound with enhanced potency.

Collectively our data are clarifying the effectiveness of the drug against its targets for further *in vivo* applications. Previous data have shown that the conjugation of the oligoarginine part increases the permeability of the attached drug in cancer cells^14^. However, once the drug reaches its target, its correct function should be clarified properly. Our attempt to obtain this piece of knowledge could be considered as an important contribution to the clarification. Thus, our current results summarised above could fill the gap in the literature of the effectiveness of the Dau-Arg_6_ against its molecular targets.
